# Supplementary material for: Individualized Prediction of SARS-CoV-2 Infection in Mexico City Municipality during the First Six Waves of the Pandemic
Source: Healthcare (Basel). 2024 Mar 31;12(7):764. doi: 10.3390/healthcare12070764 (PMC11011518; doi:10.3390/healthcare12070764)
Supplement: Supplementary file 1 [file healthcare-12-00764-s001.zip › healthcare-2894212-supplementary.pdf]

Supplementary Material

Table S1. Prevalence of symptoms (positive and negative for each wave).

Symptom	Wave 1		Wave 2		Wave 3		Wave 4		Wave 5		Wave 6	
	Positive (N = 1119)	Negative (N = 874)	Positive (N = 6372)	Negative (N = 5363)	Positive (N = 5435)	Negative (N = 7658)	Positive (N = 1150)	Negative (N = 3229)	Positive (N = 298)	Negative (N = 616)	Positive (N = 960)	Negative (N =1532)
Cough	80.8	61.8 *	51.4	22.4 *	62.5	21.3 *	48.7	14.8 *	81.1	19.3 *	63.2	54.1*
Rhinorrhea	34.4	27.0 *	27.8	9.0 *	26.7	6.5 *	28.3	11.0 *	37.4	5.4 *	41.4	35.8*
Dyspnea	46.7	26.2 *	9.9	5.1 *	8.5	3.9*	2.5	1.7	20.9	4.5 *	9.8	14.6*
Polypnea	21.4	12.1 *	3.2	1.6 *	3.1	1.1*	0.1	0.1	2.7	0.5*	3.2	4.9
Cyanosis	6.9	4.2	1.9	0.7 *	1.4	0.5*	0.1	0.1	0.7	0.2	0.9	1.9
Odynophagia	46.8	41.4	28.6	11.2 *	29.0	6.9 *	37.6	11.4 *	37.2	7.6 *	41.3	39.3
Anosmia	17.1	8.9 *	19.0	4.8 *	14.3	1.6*	2.0	1.1	2.1	0.7	4.4	2.8
Dysgeusia	17.9	8.1 *	16.8	4.6 *	12.5	1.6*	1.7	0.9	2.8	0.5 *	3.7	2.8
Diarrhea	23.6	20.9	9.6	4.6 *	11.3	6.7 *	5.7	2.8 *	10.4	4.7 *	11.0	9.9
Abdominal pain	13.9	12.3	5.3	1.8 *	7.3	3.2 *	3.3	1.5 *	17.2	3.4 *	5.9	7.3
Vomit	9.9	7.1	3.3	1.4 *	4.3	1.5*	2.0	1.1	5.4	1.9 *	3.0	6.3*
Headache	75.2	70.0 *	44.4	19.7 *	45.6	15.5 *	32.0	12.3 *	65.0	17.0 *	48.1	46.2
Myalgia	59.7	43.8 *	25.3	8.2 *	26.2	4.7 *	22.5	6.4 *	48.1	10.7 *	34.2	31.3
Arthralgia	55.7	41.3 *	21.4	6.2 *	20.8	3.6 *	13.9	3.6 *	30.0	5.9 *	27.6	23.7
Chest pain	31.4	22.0 *	14.3	6.0 *	15.4	6.2 *	7.0	2.5 *	30.3	8.6 *	16.8	16.9
Fever	71.8	45.2 *	33.4	13.0 *	42.2	10.6 *	18.3	4.2 *	52.7	9.3 *	35.3	31.3
Chills	43.8	29.1 *	21.8	6.5 *	27.5	7.2 *	22.0	5.7 *	40.7	6.7 *	28.6	26.6
Irritability	19.4	16.0	8.7	5.1 *	10.1	5.9 *	2.9	0.7 *	13.1	5.4 *	11.6	12.0
Conjunctivitis	10.9	11.3	7.3	2.2 *	8.5	2.7*	1.6	1.1	15.5	2.3 *	7.8	10.8

* $p < 0.01$ compared with positive test result of the same wave.

Table S2. Binary logistic regression analysis to predict an individual SARS-COV-2 test for each wave. Data are presented as odds ratio (95% confidence interval).

Variable	Wave 1	Wave 2	Wave 3	Wave 4	Wave 5	Wave 6
Age (years)	Eliminated	1.010 (1.007 – 1.013)	1.005 (1.002 – 1.008)	Not included	1.023 (1.012 – 1.034)#	1.014(1.009 – 1.020)
Female	0.719 (0.536 – 0.966)	Not included	0.743 (0.681 – 0.810)	Not included	Not included	Not included
Smoker	Not included	1.173 (1.017 – 1.353)	0.756 (0.642 – 0.891) &	Not included	Eliminated	Not included
Hypertension	Eliminated	Eliminated	Not included	Eliminated	Eliminated	Not included
Obesity	Not included	1.475 (1.199 – 1.815)	Eliminated	Eliminated	Eliminated	Not included
Diabetes mellitus	Eliminated	Eliminated	Eliminated	Not included	Eliminated	Not included
Contact with a suspicious case	Eliminated	0.718 (0.660 – 0.782)	Eliminated	Eliminated	Eliminated	1.318 (1.082 – 1.604) &
Cough	1.813 (1.283 – 2.560)	2.045 (1.853 – 2.256)	3.165 (2.883 – 3.475)*&	2.970 (2.479 – 3.558)*&	4.369 (2.756 – 6.926)*&	1.567(1.316 – 1.864)
Rhinorrhea	Eliminated	2.210 (1.951 – 2.503)	2.084 (1.821 – 2.384)	Eliminated	3.825 (2.302 – 6.355)	Eliminated
Dyspnea	1.493 (1.051 – 2.119)	0.806 (0.669 – 0.972) *	Eliminated	Not included	Eliminated	0.534 (0.408 – 0.700) *
Polypnea	Eliminated	0.565 (0.403 – 0.792)	Eliminated	Not included	Not included	Not included
Cyanosis	Not included	Eliminated	0.533 (0.318 – 0.892)	Not included	Not included	Not included
Odynophagia	Not included	1.498 (1.328 – 1.689)	2.266 (1.986 – 2.586) &	2.231 (1.841 – 2.704) &	Eliminated	Not included
Anosmia	Eliminated	2.528 (2.034 – 3.142)	3.799 (2.819 – 5.120)	Not included	Not included	Not included
Dysgeusia	3.706 (2.302 – 5.964)	1.413 (1.127 – 1.771) *	1.712 (1.257 – 2.332)	Not included	Not included	Not included
Diarrhea	Not included	Eliminated	0.730 (0.620 – 0.860)	Eliminated	Not included	Not included
Abdominal pain	Not included	Eliminated	0.785 (0.632 – 0.973)	Eliminated	Eliminated	Not included
Vomit	Not included	0.582 (0.413 – 0.820)	Eliminated	Not included	Not included	0.408 (0.260 – 0.639)
Headache	0.694 (0.491 – 0.982)	1.590 (1.434 – 1.762) *	1.713 (1.542 – 1.902) *	Eliminated	1.960 (1.277 – 3.006) *	Not included
Myalgia	Eliminated	Eliminated	1.871 (1.575 – 2.223)	1.292 (1.014 – 1.647)	Eliminated	Not included
Arthralgia	Eliminated	1.552 (1.334 – 1.806)	1.393 (1.148 – 1.689)	Eliminated	Eliminated	Not included
Chest pain	Eliminated	1.271 (1.079 – 1.497)	Eliminated	Eliminated	Eliminated	Not included
Fever	2.259 (1.655 – 3.083)	1.864 (1.664 – 2.089)	2.959 (2.652 – 3.301) &	1.631 (1.240 – 2.146)	3.686 (2.355 – 5.768) &$	Not included
Chills	Eliminated	1.485 (1.275 – 1.729)	1.675 (1.460 – 1.922)	1.518 (1.176 – 1.959)	1.912 (1.156 – 3.164)	Not included

Irritability	Not included	0.760 (0.628 – 0.920)	0.805 (0.679 – 0.955)	2.005 (1.089 – 3.691) &#	Eliminated	Not included
Conjunctivitis	Not included	1.309 (1.025 – 1.670)	Eliminated	Not included	Eliminated	Not included

Not included= variable not included in the regression model, Eliminated: Variable eliminated from the regression model.

* $p < 0.05$ vs wave 1, & $p < 0.05$ vs wave 2, # $p < 0.05$ vs wave 3, $ $p < 0.05$ vs wave 4, ≠ $p < 0.05$ vs wave 5.

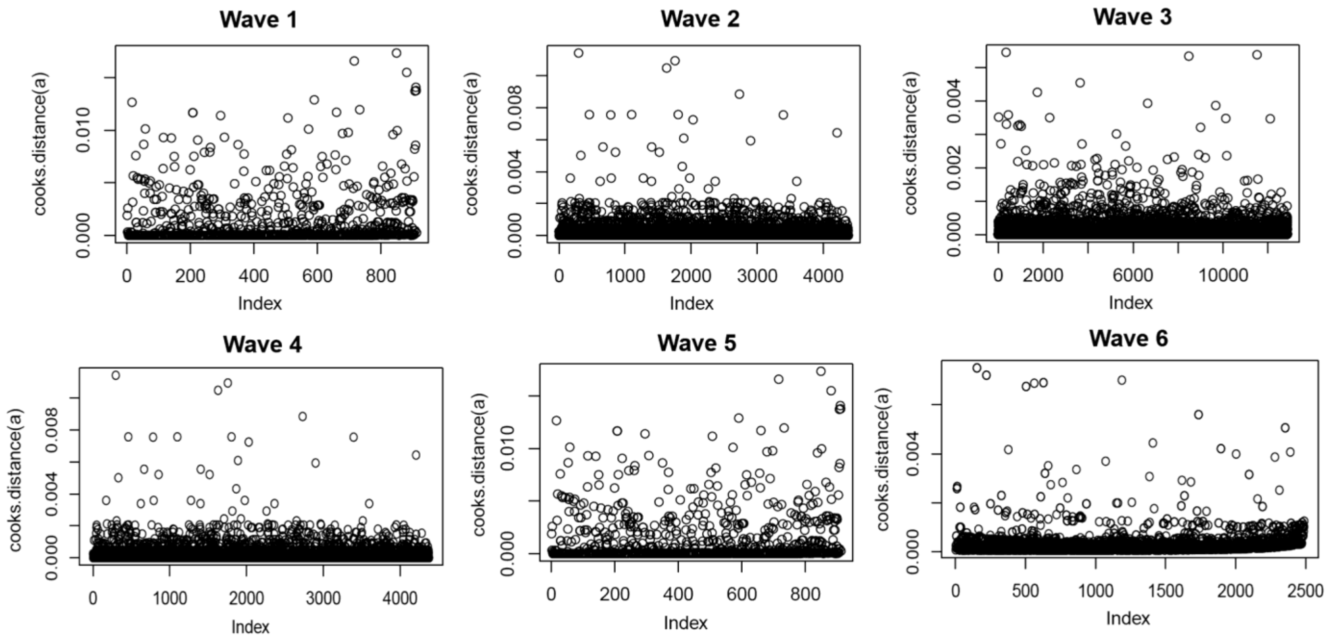

Figure S1. Cook's distance plot was calculated from each wave's final regression model to identify potential influential points.
